# Comparative Assessment of Robotic versus Classical Physical Therapy Using Muscle Strength and Ranges of Motion Testing in Neurological Diseases

**DOI:** 10.3390/jpm11100953

**Published:** 2021-09-25

**Authors:** Zoltán Zsigmond Major, Calin Vaida, Kinga Andrea Major, Paul Tucan, Emanuela Brusturean, Bogdan Gherman, Iosif Birlescu, Raul Craciunaș, Ionut Ulinici, Gábor Simori, Alexandru Banica, Nicoleta Pop, Alin Burz, Giuseppe Carbone, Doina Pisla

**Affiliations:** 1 Neurophysiology Department, National Center for Spinal Disorders, Királyhágó u. 1, 1126 Budapest, Hungary; zoltan.major@eeg-emg.ro; 2 Neurology Department, Municipal Clinical Hospital Cluj-Napoca, 400139 Cluj-Napoca, Romania; emabrusturean@yahoo.com (E.B.); raul.craciunas@yahoo.com (R.C.); simorigabor@gmail.com (G.S.); 3 Research Center for Industrial Robots Simulation and Testing, Technical University of Cluj-Napoca, 400114 Cluj-Napoca, Romania; paul.tucan@mep.utcluj.ro (P.T.); bogdan.german@mep.utcluj.ro (B.G.); iosif.birlescu@mep.utcluj.ro (I.B.); ionut.ulinici@omt.utcluj.ro (I.U.); alexandru.banica@omt.utcluj.ro (A.B.); nicoleta.pop@mep.utcluj.ro (N.P.); alin.burz@mep.utcluj.ro (A.B.); 4 Second ICU, Neurosurgery Department, Cluj County Emergency Clinical Hospital, Strada Clinicilor 3-5, 400000 Cluj-Napoca, Romania; majorkinga@yahoo.com; 5 DIMEG, University of Calabria, Via Pietro Bucci, 87036 Rende, Italy; giuseppe.carbone@unical.it

**Keywords:** robotic rehabilitation, physical therapy, stroke, Parkinson’s, motor neuron disease, dynamometry, goniometry, MRC scale

## Abstract

The use of robotic systems in physical rehabilitation protocols has become increasingly attractive and has been given more focus in the last decade as a result of the high prevalence of motor deficits in the population, which is linked to an overburdened healthcare system. In accordance with current trends, three robotic devices have been designed, called ParReEx Elbow, ParReEx Wrist, and ASPIRE, which were designed to improve upper-limb medical recovery (shoulder, elbow, forearm, and wrist). The three automated systems were tested in a hospital setting with 23 patients (12 men and 11 women) suffering from motor deficits caused by various neurological diseases such as stroke, Parkinson’s disease, and amyotrophic lateral sclerosis (ALS). The patients were divided into three groups based on their pathology (vascular, extrapyramidal, and neuromuscular). Objective clinical measures, such as the Medical Research Council (MRC) scale, goniometry, and dynamometry, were used to compare pre- and post-rehabilitation assessments for both robotic-aided and manual physical rehabilitation therapy. The results of these tests showed that, with the exception of a few minor differences in muscular strength recovery, the robotic-assisted rehabilitation methods performed equally as well as the manual techniques, though only minor improvements were validated during short-term rehabilitation. The greatest achievements were obtained in the goniometric analysis where some rehabilitation amplitudes increased by over 40% in the vascular group, but the same analysis returned regressions in the neuromuscular group. The MRC scale analysis returned no significant differences, with most regressions occurring in the neuromuscular group. The dynamometric analysis mostly returned improvements, but the highest value evolution was 19.07%, which also in the vascular group. While the results were encouraging, more research is needed with a larger sample size and a longer study period in order to provide more information regarding the efficacy of both rehabilitation methods in neurological illnesses.

## 1. Introduction

In neurological diseases, motor recovery is often deficient, with many patients experiencing long term impairments that have a negative effect on their quality of life and their capability of performing daily living activities. The increased survival of the population and chronic morbidities create the premises for a higher incidence of motor dysfunction, regardless of the lesion topography, involving either the pyramidal, extrapyramidal, or the peripheral motor system.

Physical therapy consists of specific exercises that have been developed to reestablish the functionality of the affected segment of the motor system and acts as an adjuvant to medical treatment. These movements can be easily taken over by automated systems [1] and robots, thus optimizing the frequency and time allocated to each exercise cycle, with the physical therapist only needing to configure the device with a personalized treatment protocol [2].

Previous studies have compared the results of robotic physical therapy to those of classical physical therapy.

Masiero et al. [3] analyzed the effects of the robotic rehabilitation using the NeReBot device, which is able to perform rehabilitation on patients in an acute state. The study involved 35 patients (17 in the experimental group and 18 in the control group) in the hemiparetic and hemiplegic state. The results of the study revealed a higher reduction in motor impairment and enhancements in paretic upper-limb function in the case of robotic rehabilitation due to extended amount of time that the patient spent in rehabilitation.

Singh et al. [4] conducted a randomized controlled trial to assess the evidence of neuroplasticity in robotic-assisted hand rehabilitation. The trial involved 27 patients divided into two groups: 13 patients in the robotic physical therapy and 14 in the conventional physical therapy group. Clinical scale and neurophysiological parameter improvements were recorded in the robotic therapy group with respect to the conventional therapy. These improvements were catalogued as a consequence of plastic reorganization and use-dependent plasticity.

Taravati et al. [5] conducted a study regarding the differences between robotic therapy and classical therapy. The study involved 45 patients, 22 of whom were randomly assigned to robotic therapy by a computer, with the remaining 23 patients being assigned to conventional physical therapy. The results showed similar efficacy in terms of motor function, quality of life, cognition, and emotional status; however, in terms of work power and psychological recovery, robotic physical therapy was slightly more beneficial than conventional physical therapy was.

The efficacity of robotic physical therapy was also tested in [6,7,8,9], with similar results: robotic physical therapy was as effective as conventional physical therapy.

ASPIRE, ParReEx Elbow, and ParReEx Wrist are a family of modular devices targeting the rehabilitation of the shoulder, elbow and wrist and were developed in a modular configuration with a unified, centralized, control system and a user interface. As it is a robotic system that uses a unified control system, it makes it possible to treat 1, 2, or 3 patients at the same time under the supervision of a single physician. Each robotic module is fast and easy to setup and configure. The targeted cost of the rehabilitation robotic system (all three modules) is under EUR 50,000, making its implementation in various hospitals in Eastern Europe a feasible low-cost solution, enabling a larger number of people to access rehabilitation as well as requiring a lower number of therapists needed to perform the rehabilitation, given the fact that a therapist can work with up to three patients at the same time using the robotic system.

Within this study, the validation of the robotic system through a first set of clinical trials is targeted.

The prototype of the robotic system validated through the clinical trial is only configured for the rehabilitation of the right arm: before preparing the current clinical study, an analysis regarding the hand dominance and side of stroke was completed.

In 2005 Waller et al. [10] examined the difference between left and right hemispheric lesions at baseline and after arm training. The study involved 22 patients (50% with left hemispheric lesion and 50% with right hemispheric lesion). All of the subjects were right-handed and had chronic strokes. The subjects performed a six-week repetitive bilateral training regime, and the results showed no statistical differences between the two groups. However, patients with left hemispheric lesions displayed significantly greater passive paretic wrist extension.

A 2006 study by Harris et al. [11] determined if upper extremity impairment rehabilitation depended on whether the dominant hand was affected or not. The study involved 93 patients who had suffered a stroke at least 12 months before the analysis and who had residual upper-limb impairment of the dominant hand. The study revealed that the patients with a severely or mildly impaired dominant hand presented less impairment than the patients with an affected non-dominant hand. The study also revealed that even though arm dominance impacts the impairment, neither group had demonstrated better performance in the execution of daily tasks. 

In 2014, Nam et al. [12] evaluated how the paralysis of the dominant hand impacts the quality of life of stroke survivors in the subacute state. A total of 75 patients were recruited for the study and were divided into two groups based on the location of the lesion and the side of the dominant hand (the first group contained patients with the dominant hand affected, and the second group of patients had their non-dominant hand affected). The study revealed no statistically significant difference between the two groups and that the effect of paralysis on the dominant hand in patients with subacute stroke is not significantly different compared to the effect of paralysis on the non-dominant hand.

To test and validate the performance of the previously mentioned robotic-aided physical therapy devices, an exploratory, multimodal evaluation was conducted in the Neurology Department of the Municipal Clinical Hospital Cluj-Napoca (SCMCJ). On the one hand, Neurophysiological tests were conducted, and given the extent of the obtained data, these results were separated and have already reported by the research group [13]; on the other hand, the present evaluation targeted the physical strength and the ranges of motion of the studied joints after rehabilitation, both for the exercises conducted by the therapists and by the robotic devices.

In the field of neurology, pathologies are often characterized by serious motor deficits. Motor deficits might occur either suddenly, such as in cases of cerebrovascular diseases, or gradually, such as in cases of neurodegenerative pathologies, for example, motor neuron diseases. Sometimes, motor deficit is absent, but the lack of proper regulation/modulation has a huge impact on movement quality. Starting from the above premises and by judging the most frequently occurring disease for each category, the target pathologies for the current study were ischemic stroke, Parkinson’s disease, and amyotrophic lateral sclerosis (ALS). Stroke patients have an extensive cerebral lesion and present deficiencies corresponding to that territory. Extrapyramidal diseases are characterized by degenerative lesions of the basal ganglia, and the deficiencies mainly involve inappropriate movement modulation. In motor neuron diseases, in different parts of the brain, both the central–motor cortex and the peripheral–corticospinal tracts, the peripheral motor neurons might suffer degeneration. The motor deficits are composed of an upper and lower motor neuron syndrome.

Physical rehabilitation, besides the preservation of joint integrity and direct influences on muscle trophicity, also represents an important trigger for plasticity [14]. Ischemic stroke patients benefit the most from rehabilitation [15], regardless of whether it is performed by a therapist [16] or using robotized devices [17]. Neuroplasticity and the existence of perilesional penumbra zones account for this effect of rehabilitation. For diseases of the basal ganglia, proprioceptive input probably increases the excitatory cortico-striatal influences and improves the activity of the indirect pathway, with a seemingly beneficial clinical effect [18,19]. Concerning motor neuron diseases, active recuperation appears to only have a restricted impact, which is potentially simply maintain joint mobility, and, to some extent, safeguard muscle strength for a more drawn-out period. Unfortunately, the disease is rapidly progressive, as the neurophysiologic changes overcome the capacity of recovery [20,21].

To clinically evaluate the efficiency of the rehabilitation protocols, both the movement forces and amplitudes should be evaluated. The Medical Research Council scale (MRC) is a widely accepted and validated tool for the evaluation of motor deficit. It appreciates the strength of a muscle group in a semi-quantitative manner, while goniometry and dynamometry are more objective methods that permit an exact, quantitative evaluation of neurological diseases [22,23]. The current investigation intends to assess the effects of actual restoration on the objective pathologies that are related to the equal assessment of the robotized approach in order to build proficiency and diminish expenses.

Our initial exploratory study has the following objectives: (1) to assess the benefits of physical therapy for different neuromotor disorders(this objective was described in [13]); (2) to comparatively assess robotic-assisted therapy and classical therapy applied by a physical therapist, and (3) to identify potential optimization solutions for the robotic therapy devices to improve their performance with respect to patient progress (the optimization of the robotic structures after clinical trials based on clinician opinion, patient progress, and acceptance was described in [24]).

## 2. Materials and Methods

**Figure 1:**
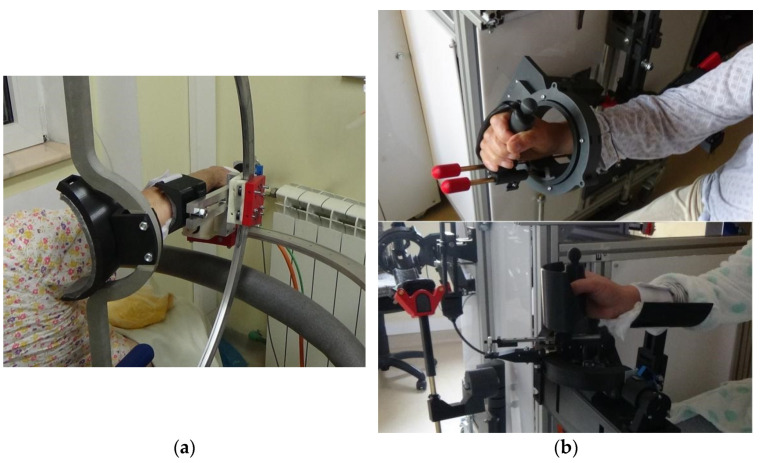
Experimental rehabilitation robots. (**a**) ASPIRE module; (**b**) ParReEx Elbow module (up); and ParReEx Wrist module (down).

The robotic systems ASPIRE [25,26,27], ParReEx Elbow, and ParReEx Wrist [28,29,30] (Figure 1) are innovative parallel robots that were designed for upper limb rehabilitation (shoulder, elbow, forearm, and wrist) and have already been presented in detail in a dedicated publication [23]. The ASPIRE (Figure 1a) robotic device has a spherical parallel structure and was designed for the rehabilitation of the shoulder joint. The device has three degrees of freedom, two of which are meant to emulate the major motions of the shoulder joint (mainly shoulder abduction/adduction and shoulder flexion/extension), with the third performing forearm pronation/supination, which is necessary to ensure the correct execution of the main shoulder motions within their complete ranges of motion (ex. flexion over 90 degrees). The ParReEx (Figure 1b) robotic device consists of two independent modules. The first module has two degrees of freedom and performs the motions of the elbow joint (flexion) and the forearm segment (pronation/supination). The second module targets the rehabilitation of the wrist joint and has two degrees of freedom (one for flexion/extension and the other for ulnar deviation and radial deviation). The devices use a single, centralized control system that allows the therapist to control each module individually from the same user interface.

The devices were developed to be adaptable to different limb characteristics, thus being able to cover a wider range of patients. Of these, there are also mechanically adaptable mechanisms to compensate for different limb segment lengths as well as the usage of textile straps, allowing limb segment girth adaptation.

### Evaluation Protocol

The study protocol was approved by the Ethics Committee of the SCMCJ. It was developed in accordance with the principles of the Declaration of Helsinki regarding biomedical research. All of the participants signed an informed consent detailing the scope and details of the medical procedure, the functionality of the robot, and the environment in which said process would take place. The duration of the study was 3 months, from October to December 2019. A total of 23 patients were admitted (12 men and 11 women). The participants showed various degrees of upper limb motor deficit.

Inclusion criteria sought patients within the age range of 18–80 years; suffering from ischemic/hemorrhagic stroke, Parkinson’s disease or, ALS; having suffered a neurological disorder within the last 2–24 months; have a mini-mental scale(MMS) score of 18–23 (mild cognitive impairment) or 24–30 (no cognitive impairment), as long as the mild cognitive impairment does not influence the patient’s capability to perform the physical therapy; be Brunnstrom stage (BS) 3 (voluntary synergy movements, producing movement across joints, increased spasticity), 4 (voluntary movements outside of synergy patterns, decreasing spasticity), 5 (developing control of individual or isolated movements), or 6 (return to near-normal motor control); have a modified Ashworth scale (MAS) score of 1 (slight increase in muscle tone, manifested by a catch and release or by minimal resistance at the end of the ROM (range of motion) when the affected limb is moved in flexion or extension), 1+ (slight increase in muscle tone, manifested by a catch followed by a minimal resistance throughout the remainder of the ROM), or 2 (more marked increase in muscle tone throughout most of the ROM, but affected part easily moved); and have left arm impairment, right arm impairment, or bilateral impairment.

Inclusion criteria regarding rehabilitation with the robotic system (along with the ones above) required the patient to present right arm impairment (shoulder, elbow, or wrist), be able to maintain a sitting position without any aiding devices other a than wheelchair, be of a stable state (able to understand the functionality of the robot and provide informed consent regarding the procedure), and have no wounds on the surface of the right arm(wounds may interfere with the anchor points and create pain for the patient, influencing the effect of the therapy).

The exclusion criteria were minor patients or patients older than 80 years, health conditions that were incompatible with the physical therapy (respiratory deficit, hearth conditions, severe spasticity, paralysis, spasms, severe cognitive impairment), social constraints (patient denied the fact that he needed therapy, patient avoided medical control and doubted the effects of the rehabilitation, or conflict—family or relatives of the patients advised against rehabilitation), and economic constraints (the patient was unable to access funds from the health minister for hospitalization or he was not enrolled with any health provider). Exclusion criteria regarding rehabilitation with the robotic system implied left arm impairment, the patient being unable to maintain sitting position, the unstable state of the patient (patient was unable to understand that he was going to perform rehabilitation with a robot instead of a human), or the patient presenting wounds or cannula on the right arm.

The participants were grouped according to their pathology, resulting in a Vascular, an Extrapyramidal, and a Neuromuscular group.

**Figure 2:**
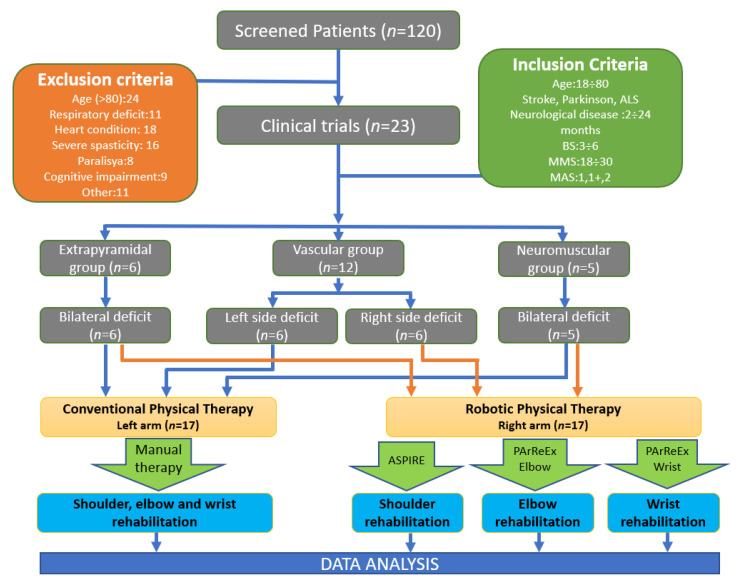
The setup of the clinical exploratory study with the patient distribution between manual and robotic therapy.

The selection and distribution of the patients between the manual and robotic therapy is illustrated in Figure 2. The first group (Vascular) was formed by 12 patients, six women and six men, with ischemic stroke and with upper limb deficit. Right-side deficits were treated by robotic therapy, and left-side deficits were treated by the physical therapist; six patients had a right-side deficit (three women, three men), and six patients had a left-side deficit (also three women and three men). The second group consisted of three women and three men with Parkinson’s disease, and these patients were designated to the Extrapyramidal group. While a slight asymmetry between the two sides is present at the beginning of the disease, the dysfunctions are bilateral. For this reason, each patient was its own control, with the right side being treated using the robotic system and the left side being treated using classical physical therapy. The third group was formed by five patients, two women and three men, all of whom had motor neuron disease. The same principle was used in this case as well: the right side was treated using the rehabilitation devices, while the left side benefited from exercises performed by the therapist. The protocol was the same for patients receiving robotic physical therapy and for the ones receiving conventional physical therapy.

Table 1 presents the rehabilitation protocol used for robotic rehabilitation and for conventional rehabilitation (which is identical).

The time spent by each of the patients enrolled in the trial was 15 ± 2 min with the ASPIRE system for shoulder rehabilitation, 15 ± 3 min with the PArReEx elbow system for elbow rehabilitation, and 10 ± 2 min with the PArReEx Wrist system for wrist rehabilitation. Each patient preformed two rehabilitation sessions daily. Conventional rehabilitation was performed by the physiotherapist with the patient seated on the side of a hospital bed, and the entire rehabilitation motion was performed manually by the physiotherapist. Times recorded during the conventional rehabilitation were lower (12 ± 1 min for shoulder rehabilitation, 11 ± 2 min for elbow rehabilitation, and 8 ± 1 min for wrist rehabilitation).

The amount of the physical therapy delivered for the left hand was identical to the therapy delivered for the right hand.

When joining the study, each patient was informed of the robotic system and saw the robotic system working, had time to individually evaluate the potential benefits, and signed an informed consent. A baseline clinical, gonio-, and dynamometric evaluation was performed. Goniometry evaluates the motion ranges of each assessed joint, and the gathered data are expressed in degrees. The serial investigation provides information regarding the possible gain in joint flexibility after seven consecutive days of physical therapy, which is important for both the spastic and plastic hypertonic states, such as in our patient groups. Below are the exact measurement protocols for each investigated joint.

For the measurements, the patients should ideally be at rest in a standing position, with the upper limb in supine position next to the body. Under the conditions of the investigated pathologies, this is not possible, so each measurement tends to be as close to the resting position as possible but is adapted to the individual medical condition.

**Figure 3:**
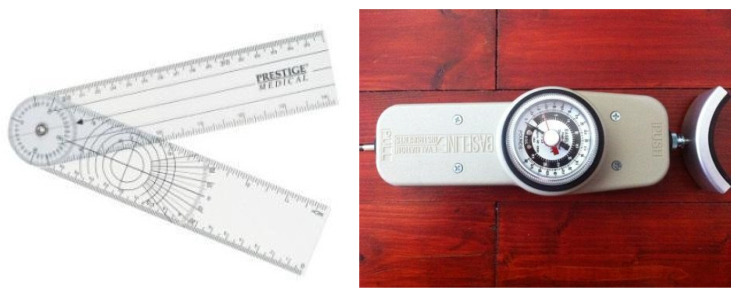
The standard analogue goniometer and baseline push–pull dynamometer used for the measurements.

For shoulder flexion, the center of the goniometer (Figure 3) is fixed at the center of the scapulo-humeral joint with the arms parallel to the arm. The patient flexes the upper limb; meanwhile, the fixed arm of the goniometer remains parallel with the body, and the mobile arm moves together with the humerus. The formed angle is the shoulder flexion value. For shoulder extension, the method is similar, except the patient executes arm extension. The shoulder abduction and adduction start from a different premise, with the arms of the goniometer positioned to be parallel with the coronal plane of the body. Then, the measurement takes the same course, and the mobile arm follows the humerus.

Elbow flexion is measured with the upper limb in 90-degree abduction while keeping the hand, elbow, and shoulder in the same plane during the movement. The center of the goniometer is positioned on the olecranon, the fixed arm is kept parallel to the humerus, and the mobile arm is kept parallel to the ulnaris bone. The patient executes forearm flexion on the arm, with the measured angle being in the range of motion for elbow flexion. In cases where elbow flexion is being measured, the degrees of freedom also permit pronation and supination. With the elbow next to the torso and in 90-degree flexion and with the interior face of the forearm oriented medially, the patient holds a wooden stick in his/her hand. The arms of the goniometer are positioned parallel to the stick. Then, the patient executes the movement, the fixed arm is kept in position, the mobile arm follows the movement, and the resulting angles are registered as ranges of motion for pronation/supination.

The flexion and extension of the radio-carpal joint was evaluated in the present study. For both radio-carpal flexion and radio-carpal extension, the center of the goniometer is positioned on the lateral epicondyle of the ulna, the fixed arm of the goniometer is parallel to the ulna, and the mobile arm moves together with the hand. More detailed information and imagery is described in the literature [31].

Muscle strength is another parameter being investigated in the current study, with motor deficit being the most important factor for vascular and neuromuscular diseases. It is frequently measured for each movement. For this purpose, to reduce the subjectivism of the evaluation, measurement scales were developed. This study uses such a scale, with the Medical Research Council scale (MRC) being used in the current study, which is reproduced in Table 2.

Using this technique, one of the investigators performed all of the examinations as follows: abduction, adduction, flexion, and extension of the shoulder; elbow flexion and extension; flexion and extension of the hand; supination and pronation of the forearm; finger extension of the metacarpal-phalangeal and inter-phalangeal joints; and finger adduction and abduction. The measurements were performed on a wider number of joints than those included in the rehabilitation protocol in order to study the influence of the exercises on other joints of the upper limb. The other, more objective method for the evaluation of muscle strength is dynamometry. This is a process that uses a specialized device—in the present study, a Baseline Push-Pull Dynamometer—to measure quantifiable data for muscular force (expressed in kgf), power, and resistance. As with goniometry, in this case, the positioning of the patient, or the limb and the limb segment is incredibly important in order to obtain reproducible data.

Strength during shoulder flexion is preferably measured on sitting patients by positioning the dynamometer on the distal third of the antero-medial surface of the arm, with the shoulder being stabilized by the therapist. The recording starts with the flexion of the elbow against the resistance of the therapist and the in-between device. To measure the strength during shoulder extension, the patient position is similar; there is a need to stabilize the shoulder, and the dynamometer should be put on the distal third of the arm but this time on the postero-lateral surface. The measurement starts with the extension of the shoulder. Strength measurements during shoulder abduction and adduction are performed similarly, except the dynamometer should be positioned on the lateral or medial surface of the distal third of the arm, and the performed movement should be executed accordingly.

To measure strength during elbow flexion, the body is still in a sitting position, the shoulder is flexed 45 degrees, and the elbow is flexed at 90 degrees with the palm facing upwards. The therapist stabilizes the arm and places the dynamometer on the distal third of the forearm medially and maintains resistance against the flexion performed by the patient. The same is steps are performed to measure the strength during extension, but the device should be positioned on the lateral surface of the forearm, and the data recording is made during extension. Measuring strength during elbow pronation and supination requires the same patient position, but the subject is asked to hold a wooden stick in his/her hand. The dynamometer is placed on the lateral part of the stick, and the patient is asked to perform the movement.

Measuring the radio-carpal joint flexion/extension strength requires the same position from the patient, but the forearm must be stabilized by the therapist. Then, for the measurement, the dynamometer is placed either on the palmar or on the outer side of the hand and the appropriate movement is performed against the therapists’ resistance.

Regardless of the deficit, the muscles to be tested need to be isolated by stabilizing the patients’ position in a personalized manner; the angle of different joints should be chosen in a way that is suitable to start a particular movement. Even if optimal, the measurements should be repeated three times; if they are not quantifiable, then after a few minutes, the whole procedure should be repeated; it is useful to start on the unaffected side first. The evaluation is correctly executed, reproducible, and comparable if it is always performed by the same examiner. All of the baseline measurements were conducted by the same investigators/method in order to further reduce the bias induced by subjectivism. After the baseline, a 7 day long, 2 times/day rehabilitation program was implemented (Table 1), and then the presented evaluation was performed again at the end of the rehabilitation.

The obtained data were gathered in a database, and afterwards, it was statistically analyzed using IBM SPSS Statistics 20 (IBM Corp. Released 2011. IBM SPSS Statistics for Windows, Version 20.0. Armonk, NY, USA: IBM Corp.). After the descriptive statistics were determined, the chosen analysis was non-parametric given the low number of participants. Since the data rows constituted their own control for the second and third groups, the Wilcoxon Matched Pairs test was used. An exception was the Vascular group, where the control treated by the physical therapist was an individual group with left hemiparesis. In this case, the test used for the analysis was the Mann–Whitney U test for independent variables. Each data row for every individual parameter was tested for the type of distribution using the Kolmogorov–Smirnoff test, and since normal distribution was found, the best evaluation method was applied. The significance threshold was considered *p* < 0.05.

## 3. Results

Each test was performed at the beginning and at the end of the rehabilitation process, with the first test representing the baseline clinical evaluations.

Demographic data and some characteristics of the patients enrolled in the clinical trials are shown in Table 3.

The assessment of the patients started with goniometry, which was expressed in degrees. The results were compared with the baseline assessment of the patients. The results are presented in Table 4, with most improvements being visible in the Vascular group where the ROM of the shoulder extension and adduction improved by more than 40%. Within the extrapyramidal group, the biggest improvements were recorded in the elbow pronation and radio-carpal extension. The patients in the neuromuscular group recorded few improvements, with the maximum amount of improvement demonstrated in the elbow supination (6.05%). Some slight regressions in shoulder abduction were also recorded in the Vascular group, and shoulder flexion and radio-carpal flexion regressions were demonstrated in the neuromuscular group.

The evolution of the MRC data with respect to the baseline are presented in Table 5. The biggest improvements in the case of the Vascular group are in shoulder extension and elbow flexion. In the Extrapyramidal group, the biggest improvements were demonstrated in terms of shoulder flexion and elbow pronation. The Neuro muscular group recorded no significant improvements; however, there were some regressions in this group in terms of shoulder and radio-carpal motion. The Vascular group also recorded regressions in radio-carpal motion.

The results of the dynamometric analysis are presented in Table 6. The Neuromuscular group recorded slight improvements in elbow flexion and pronation and radio-carpal flexion and extension. The Extrapyramidal group recorded the biggest improvements in terms of radio-carpal flexion and extension. The Neuromuscular group recorded slight improvements in shoulder flexion and radio-carpal extension Regressions were also recorded, but this time, the differences were almost insignificant compared to the regressions recorded in previous assessments (goniometry and MRC).

Table 4, Table 5 and Table 6 make it easier to understand the differences between the baseline assessment and the final evaluation; however, from a statistical point of view, the null hypothesis is validated when analyzing the *p*-values of each pathology group and each rehabilitation motion. In order for the null hypothesis to be accepted, no significant difference the *p*-values must score above the previously defined threshold (*p* > 0.05). Table 7 presents *p*-values for each group and rehabilitation motion, even if there were differences recorded between baseline assessment and final evaluation in terms of percentage, there was no *p*-value that scored under 0.05.

## 4. Discussion

The results presented above offer a preliminary assessment regarding functional changes following the rehabilitation process in the studied pathologies. The results are discussed from the premise of not being statistically significant, with this being a consequence of either the low number of investigated cases or the reduced duration of the rehabilitation process. The discussion considers tendencies in order to sketch possible evolutions at an exploratory level. In the below discussion, the obtained data are grouped according to the evaluated pathologies.

Ischemic stroke produces a typical lesion pattern characterized by the death of directly affected cell groups located in the middle of the affected area and by the suffering but potential survival of cells at the outer limit of the lesion, the penumbra area. Under favorable circumstances, this region might regain function, sometimes leading to spectacular recovery. According to one trend, commencing physical rehabilitation as quickly as possible after the patient is table frequently produces quite visible effects. The physiopathology is probably proprioceptive signaling and primary somatic-sensory to motor cortex triggering, and this enhances neuroplasticity [31].

As our study showed, there was no difference in terms of whether the rehabilitation was conducted using robotized or therapist-implemented protocols.

The baseline assessment revealed lower ROM in the Vascular group, where the biggest improvement was also recorded, but the ROM of the Extrapyramidal group and the Neuromuscular group were still higher than the ROM of the Vascular group, even after the rehabilitation. There were no significant differences between the groups regarding the MRC scale and dynamometry, either in baseline or at the final assessment.

*Vascular group*: the effect of rehabilitation on this group is quantifiable using the proposed methods, as demonstrated for the selected pathology by several studies [32,33,34]. The MRC value for a given movement increases in parallel with clinical recovery. This relationship is also verified in the present study, most noticeably in the vascular group. This increase is visible for each evaluated muscle and movement, in accordance with the literature, despite being statistically non-significant. The latter effect is probably given by the mentioned limitations of our study. The dynamometric evaluation eliminates the subjective component of MRC evaluation when properly executed—same technique, same investigator. The results show similar overall trends, with a more reduced extent than MRC, except for in the distal joints, where a post-rehabilitation decrease was observed. This raises the question of if there is any difference in the effect of rehabilitation on the different pathways given the fact that the motor control of the proximal and distal upper limb is provided by different descending paths. The dorsolateral tracts (e.g., the corticospinal and rubrospinal tract) are important for distal movements, and the ventromedial tracts (reticulospinal, vestibulospinal, and tectospinal tracts) are important for the muscles and axial and proximal movements; however, there is the potential that this effect is that result of inconsistent data in this portion of the study.

Goniometric evaluation of an ischemic stroke patient makes sense when spasticity is already installed or is in course of being installed in order to alleviate its effect on the patient’s movements and ranges of motion. Our recruited patients were subacute to chronic patients, making them suitable for the evaluation. The approach holds quite a well-defined methodology. The evaluation being conducted by the same evaluator is a necessary condition to preserve objectivity. Rehabilitation led to a slight increase in the ranges of motion at the investigated joints, underlining an otherwise proven effect of physical therapy on spasticity.

*Extrapyramidal group*: in this case, one cannot speak about motor deficit; the symptom is rather definable and is motor dysfunction resulting from the lack of or disturbed modulation of motor activity. Both the direct and indirect pathways involved in Parkinson’s disease led to reduction of the excitatory feed-back loop on the motor cortex. This result is produced via the direct pathway by the lack of D1 receptor signaling on the globus pallidus internus (GPi) and the consequent reduction of ventro-lateral and ventro-anterior thalamic nuclei excitatory influences on the motor cortex [35]. A similar reduction of excitatory signaling is seen when dopamine reduction via D2 signaling reduces the inhibition of the globus pallidus externus (GPe), which, in turn, releases the sub-thalamic nucleus, stimulating the GPi and inhibiting the mentioned thalamic nuclei. Recent research has demonstrated that physical exercises have clear benefits in PD and should be included in the long-term patient management [36].

When compared to the other investigated pathologies, the Extrapyramidal group showed clear, consequent movement towards an increase in all of the investigated parameters. This increase is not significant and is probably caused by the mentioned limitations; still, the visual tendency is obvious. The most pronounced effect is seen for the MRC scale evaluation, and the least modified parameter is the dynamometric assessment. Physical strength theoretically remains the same, but the correct allocation of motor resources leads to more increased peripheral activation. The ranges of motion were expected to show a tendency towards an increase by means of a reduction in the rigidity and plastic hypertonia. The proprioceptive input increased the excitatory cortico-striatal influences, which, in turn, in the relative absence of dopamine, improve the activity of the indirect pathway, inhibiting the thalamic excitatory loop. The overall effect seems to be beneficial in the short term; *however, the long-term effects have not been evaluated* [37].

*Neuromuscular group*: The whole motor system is affected by the degeneration process, including the upper and lower motor neurons and the in between pathways, leading to muscle atrophy and mixed motor deficit. Through clinical testing, both muscle strength and goniometry show disappointing results when compared to the results seen in the other pathologies. The increase in the muscle strength is only seen for a few distal muscle groups in the dynamometric measurements, which were not proved by the MRC testing, where there is not even a visual tendency towards improvement. Moreover, when investigating joint mobility, the post-rehabilitation data are even less favorable than the baseline data. The stimulating effect of rehabilitation is not beneficial centrally, at least theoretically, considering the already over-activated, excitotoxic effect of glutamate, which has been previously described in motor neuron diseases. It seems that the peripheral atrophic muscles and the individual extensively affected unstable muscle fibers show exhaustion and cell death rather than regaining strength after rehabilitation. The effect of physical therapy might be limited to the preservation of joint flexibility and to the maintenance of actual muscle performance without any performance improvement tendencies, which might overwhelm the affected motor systems. Even though the effects are limited, other clinical studies have also illustrated slight improvements in the quality of life of patients with neuromuscular disorders [38], motivating further efforts in this direction.

As mentioned at the beginning, the present study has several limitations at this stage. First, there were no long-term monitoring evaluations; thus, it was not possible to determine the long-term beneficial effects, even for short-term physical therapy. Secondly, this study had a modest sample size. Third, the duration was short, so it is not possible to make any unequivocal conclusions about the efficacy of the method, but surely completing long-term robotic/physical therapeutic rehabilitation programs can lead to clear favorable results. On the other hand, as the current study presented short, exploratory research, it provides the possibility to fine-tune the robotic systems and to set new goals for more thorough clinical research, which will include a larger number of patients.

The results of our study have also been confirmed also in a systematic review and meta-analysis [39] that assesses data from multiple clinical studies and in [30], a review that demonstrates, using clinical data, a potential paradigm change in rehabilitation therapy where robotic systems play a central role.

With respect to the defined objectives of this exploratory study, our results point out the following:

(1) *General benefits of physical therapy in neuromotor diseases*: besides the Vascular group, which is known to benefit from physical therapy, there are also promising results in the other study groups that could improve the quality of life of chronic patients and delay the effect of degenerative diseases;
(2) *Comparative assessment of robotic versus manual physical therapy*: our results illustrate that robotic therapy can be used for the treatment of neuromotor diseases with similar results as manual therapy, which, in the face of the upcoming crisis caused by the ageing of the population, represents a valid option to enable the treatment of more patients requiring physical therapy;
(3) *Possible improvements of the robotic devices*: each module of the upper limb robotic system has been carefully analyzed based on clinical data, and our team is working on improving the overall system. For the shoulder rehabilitation device, ASPIRE, our team has recently completed processing the clinical data [31], while ParReEx is expected to be ready in the coming months.

Additionally, in terms of structural improvements, the results of this clinical study provided several areas of improvement for the modular robotic system, which will aim to increase the efficiency with respect to the medical act of providing rehabilitation:

- The addition of supplementary stimuli [31], such as virtual or augmented reality interactive games [40] aiming to increase patient motivation and active involvement in the rehabilitation process;
- The improvement of the graphical user interface to investigate the possibility of working with more patients simultaneously under the supervision of a single therapist;
- Data recording using different sensors [31,40], both systemic and task oriented, which could lead to the identification of specific types of exercises for different disorders which, in turn, can lead to faster recovery and better overall outcomes.

Statistically there was no difference between the robotic rehabilitation and conventional rehabilitation, as the *p*-values obtained in this study revealed the validation of the null hypothesis on a medium-sized sample of patients. However, this result does not mean that a clinical difference is not achievable. As the percentual comparison of Table 4, Table 5 and Table 6 revealed, there were improvements and regressions (a few) in both types of rehabilitation, but the clinical importance of robotic rehabilitation is obvious give then fact that a robotic system that can perform rehabilitation equally as well as a physiotherapist, a quality that may help to overcome the lack of physiotherapists and may, at the same time, provide better access to rehabilitation for patients in order for them to receive rehabilitation at the best possible time. 

## 5. Conclusions

This initial exploratory study conducted on a group or patients with different neuromotor diseases comparatively assessed the use of robotic and manual physical therapy for rehabilitation. Our study demonstrated that there are no relevant differences between physical therapy performed by robotic devices and the conventional physical therapy that is manually performed by a therapist, encouraging future work and a more extensive clinical study. The clinical approach—MRC, gonio-, and dynamometry—remains a useful and valid technique to evaluate the results of rehabilitation and follow-up. Short term physical therapy only shows a tendency towards a quantifiable effect on the investigated pathologies, and even this is not always positive; it raises the necessity of only recommending exercises that have been carefully evaluated on physio pathological grounds.

For future work, there is a need to extend the number of participants and the duration of the study in order to be able to formulate conclusions based on significant differences and not only tendencies.

## Figures and Tables

**Table 1:** Rehabilitation protocol for each group [13].

Vascular Disease	Extrapyramidal Disease	Neuromuscular Disease
Passive motions, 2 sessions/day 10–12 repetitions: (Conventional and robotic therapy)	Passive motions, 2 sessions/day 10–12 repetitions: (Conventional and robotic therapy)	Passive motions, 2 sessions/day 8–10 repetitions: (Conventional and robotic therapy)
Radio-carpal joint flexion and extension Radio-carpal joint rotation Forearm supination and pronation Elbow flexion Shoulder flexion and extension Shoulder adduction and adduction	Radio-carpal joint flexion and extension Radio-carpal joint rotation Forearm supination and pronation Elbow flexion Shoulder flexion and extension Shoulder adduction and adduction	Radio-carpal joint flexion and extension Radio-carpal joint rotation Forearm supination/pronation Elbow flexion with 10–20% resistance, stretching program, positioning in extension Shoulder adduction and adduction

**Table 2:** MRC scale.

	MRC Scale for Muscle Power
0	No muscle contraction is visible.
1	Muscle contraction is visible but there is no movement of the joint.
2	Active joint movement is possible with gravity eliminated.
3	Movement can overcome gravity but not resistance from the examiner.
4	The muscle group can overcome gravity and move against some resistance.
5	Full and normal power against resistance.

**Table 3:** Patient demographics.

	Vascular	Extrapyramidal	Neuromuscular
Age [years] [10]	75.92 ± 1.77	71.17 ± 4.13	66.00 ± 3.85
M	6 (50%)	3 (50%)	3 (60%)
F	6 (50%)	3 (50%)	2 (40%)
Left side deficit	6 (50%)	6 (100%)	5 (100%)
Right side deficit	6 (50%)	6 (100%)	5 (100%)
Dominant hand	L 2 (16.66%)	L 2 (33.33%)	L 1 (20%)
	R 10 (83.34%)	R 4 (66.67%)	R 4 (80%)
MMS score	26.52 ± 1.283	27.11 ± 1.304	26.79 ± 1.391
Time from neurological disease [months]	9.08 ± 5.07	12.33 ± 6.54	9.20 ± 4.35

**Table 4:** Baseline and final ROM for each group.

Motion	Vascular (Degrees)		Result (%)	Extrapyramidal (Degrees)		Result (%)	Neuromuscular (Degrees)		Result (%)
	Baseline	Final		Baseline	Final		Baseline	Final	
Shoulder Flexion	82.04 ± 3.40	93.34 ± 4.42	13.77	109.11 ± 7.11	121.42 ± 3.55	11.28	107.31 ± 10.2	92.11 ± 1.21	−14.16
Shoulder Extension	34.51 ± 5.31	49.31 ± 2.75	42.89	42.63 ± 6.12	43.44 ± 4.45	1.90	44.18 ± 4.56	45.66 ± 5.65	3.35
Shoulder Abduction	59.12 ± 6.78	54.24 ± 2.38	−8.25	61.24 ± 3.24	64.16 ± 3.21	4.77	61.37 ± 3.12	63.32 ± 2.77	3.18
Shoulder Adduction	32.73 ± 3.15	48.13 ± 4.33	47.05	63.15 ± 3.14	71.84 ± 2.55	13.76	50.64 ± 2.64	51.56 ± 8.22	1.82
Elbow Flexion	121.16 ± 4.32	132.07 ± 8.22	9.00	138.21 ± 6.98	140.76 ± 8.2	1.85	137.84 ± 4.24	138.21 ± 9.98	0.27
Elbow pronation	62.36 ± 4.11	71.17 ± 5.24	14.13	72.79 ± 4.37	84.32 ± 5.12	15.84	78.10 ± 3.51	80.65 ± 6.24	3.27
Elbow Supination	63.95 ± 6.98	77.67 ± 2.22	21.45	81.34 ± 5.15	84.54 ± 4.22	3.93	81.67 ± 1.61	86.61 ± 2.55	6.05
Radio-carpal flexion	41.24 ± 4.35	49.22 ± 2.35	19.35	58.36 ± 4.61	64.13 ± 2.28	9.89	58.46 ± 7.21	57.31 ± 5.36	−1.97
Radio-carpal extension	40.61 ± 3.21	50.45 ± 4.78	24.23	52.47 ± 2.07	59.77 ± 6.32	13.91	58.22 ± 5.23	58.65 ± 4.12	0.74

**Table 5:** Baseline and final MRC scale for each group.

Motion	Vascular		Result (%)	Extrapyramidal		Result (%)	Neuromuscular		Result (%)
	Baseline	Final		Baseline	Final		Baseline	Final	
Shoulder Flexion	4.04 ± 0.21	4.12 ± 0.70	1.98	4.41 ± 0.23	4.94 ± 0.01	12.02	4.06 ± 0.15	3.95 ± 1.01	−2.71
Shoulder Extension	3.91 ± 0.13	4.32 ± 0.33	10.49	4.35 ± 0.31	4.67 ± 0.13	7.36	4.45 ± 0.23	3.93 ± 0.52	−11.69
Shoulder Abduction	4.22 ± 0.60	4.43 ± 0.12	4.98	4.74 ± 0.12	4.97 ± 0.01	4.85	4.16 ± 0.36	4.06 ± 0.36	−2.40
Shoulder Adduction	4.27 ± 0.33	4.41 ± 0.21	3.28	4.83 ± 0.08	4.94 ± 0.02	2.28	4.19 ± 0.44	4.15 ± 0.18	−0.95
Elbow Flexion	3.73 ± 0.90	4.39 ± 0.33	17.69	4.75 ± 0.10	4.92 ± 0.02	3.58	4.21 ± 0.18	4.29 ± 0.16	1.90
Elbow pronation	4.18 ± 0.60	4.47 ± 0.44	6.94	4.46 ± 0.12	4.93 ± 0.05	10.54	4.08 ± 0.22	4.08 ± 0.25	0.00
Elbow Supination	3.72 ± 1.10	3.92 ± 0.99	5.38	4.48 ± 0.40	4.76 ± 0.10	6.25	3.81 ± 0.91	3.83 ± 0.39	0.52
Radio-carpal flexion	4.19 ± 0.56	3.71 ± 1.01	−11.46	4.75 ± 0.13	4.81 ± 0.16	1.26	4.16 ± 0.28	3.95 ± 0.46	−5.05
Radio-carpal extension	3.92 ± 0.81	4.23 ± 0.22	7.91	4.78 ± 0.09	4.87 ± 0.10	1.88	4.17 ± 0.39	3.95 ± 0.38	−5.28

**Table 6:** Baseline and dynamometric evolution for each group.

Motion	Vascular (kgf)		Result (%)	Extrapyramidal (kgf)		Result (%)	Neuromuscular (kgf)		Result (%)
	Baseline	Final		Baseline	Final		Baseline	Final	
Shoulder Flexion	4.05 ± 0.25	4.03 ± 1.12	−0.49	6.11 ± 0.66	6.42 ± 0.25	5.07	4.23 ± 1.01	4.83 ± 1.03	14.18
Shoulder Extension	4.14 ± 0.38	4.15 ± 0.87	0.24	5.72 ± 0.29	5.86 ± 0.55	2.45	4.42 ± 0.28	4.54 ± 0.78	2.71
Shoulder Abduction	3.92 ± 0.68	3.91 ± 1.11	−0.26	5.98 ± 0.27	6.14 ± 0.24	2.68	4.39 ± 0.55	4.45 ± 0.88	1.37
Shoulder Adduction	3.96 ± 0.17	4.04 ± 0.88	2.02	5.91 ± 0.34	6.19 ± 0.35	4.74	4.46 ± 0.98	4.53 ± 0.47	1.57
Elbow Flexion	5.14 ± 1.20	6.12 ± 0.16	19.07	7.81 ± 0.10	7.91 ± 0.08	1.28	5.93 ± 0.19	6.31 ± 0.98	6.41
Elbow pronation	2.44 ± 1.80	2.72 ± 1.25	11.48	3.87 ± 1.12	4.24 ± 1.02	9.56	2.70 ± 1.97	2.77 ± 1.12	2.59
Elbow Supination	5.47 ± 0.98	5.52 ± 1.16	0.91	7.76 ± 0.11	7.85 ± 0.02	1.16	5.81 ± 0.88	6.06 ± 0.28	4.30
Radio-carpal flexion	2.86 ± 1.30	3.29 ± 1.23	15.03	3.93 ± 0.99	4.73 ± 0.25	20.36	2.67 ± 1.21	2.65 ± 1.09	−0.75
Radio-carpal extension	4.17 ± 0.89	4.64 ± 0.88	11.27	3.22 ± 0.94	4.16 ± 0.18	29.19	4.14 ± 1.71	4.63 ± 0.85	11.84

**Table 7:** *p*-values for each group.

Motion	Vascular Group			Extrapyramidal Group			Neuromuscular Group		
	Goniometry	MRC	Dynamometry	Goniometry	MRC	Dynamometry	Goniometry	MRC	Dynamometry
Shoulder Flexion	0.15	0.43	0.57	0.59	0.97	0.35	0.42	0.44	0.69
Shoulder Extension	0.71	0.35	0.46	0.46	0.25	0.21	0.32	0.33	0.13
Shoulder Abduction	0.18	0.84	0.87	0.71	0.27	0.35	0.37	0.68	0.47
Shoulder Adduction	0.36	0.81	0.48	0.98	0.61	0.68	0.73	0.44	0.76
Elbow Flexion	0.68	0.20	0.30	0.37	0.40	0.45	0.16	0.46	0.48
Elbow pronation	0.36	0.72	0.67	0.29	0.86	0.74	0.73	0.45	0.23
Elbow Supination	0.81	0.59	0.75	0.83	0.83	0.56	0.74	0.70	0.14
Radio-carpal flexion	0.70	0.42	0.60	0.68	0.49	0.14	0.26	0.82	0.53
Radio-carpal extension	0.48	0.36	0.39	0.34	0.69	0.20	0.48	0.54	0.48

## Data Availability

The data presented in this study are openly available in reference number [13,25,26,28].
